# Beneficial effects of anti-apolipoprotein A-2 on an animal model for coronary arteritis in Kawasaki disease

**DOI:** 10.1186/s12969-022-00783-7

**Published:** 2022-12-22

**Authors:** Fuyu Ito, Toshiaki Oharaseki, Daisuke Tsukui, Yoshitaka Kimura, Tamiko Yanagida, Fukuko Kishi, Yoshio Yamakawa, Yosuke Kameoka, Shoichi Suzuki, Kazuko Uno, Osamu Suzuki, Noriko N. Miura, Naohito Ohno, Kei Takahashi, Hajime Kono, Kazuo Suzuki

**Affiliations:** 1 Asia International Institute of Infectious Disease Control and General Medical Education and Research Center, Teikyo University, Kaga 2-11-1, Itabashi-ku, Tokyo, 173-8605 Japan; 2 Department of Pathology, Toho University Ohashi Medical Center, Ohashi 2-17-6, Meguro-ku, Tokyo, 153-8515 Japan; 3 Department of Internal Medicine, Teikyo University School of Medicine, Kaga 2-11-1, Itabashi-ku, Tokyo, 173-8605 Japan; 4 Department of Research and Development, A-CLIP Institute, Inohana 1-8-15, Chuo-ku, Chiba City, Chiba 260-0856 Japan; 5 Louis Pasteur Center for Medical Research, Tanaka Monzencho 103-5, Sakyo-ku, Kyoto, 606-8225 Japan; 6 Laboratory of Animal Models for Human Diseases, National Institutes of Biomedical Innovation, Health and Nutrition, Saito-Asagi 7-6-8, Ibaraki City, Osaka 567-0085 Japan; 7 Laboratory for Immunology of Microbial Products, School of Pharmacy, Tokyo University of Pharmacy and Life Science, Horinouchi 1432-1, Hachioji, Tokyo 192-0392 Japan; 8 Research Institute of Disaster Medicine, University1-8-1 Inohana, Chuo-ku, Chiba, Chiba 260-8670 Japan

**Keywords:** Kawasaki disease, Anti-apolipoprotein A-2, Vasculitis, Coronary arteritis

## Abstract

**Background:**

Kawasaki disease (KD) is usually treated with high-dose intravenous immunoglobulin (IVIg) as severe infectious and other diseases. Due to issues that are associated with immunoglobulin preparation, such as the risk of possible contamination by infectious agents and limited blood banking resources, recombinant immunoglobulins are required.

We developed a novel recombinant antibody drug candidate, “VasSF,” based on the therapeutic effects it exerted on a mouse spontaneous crescentic glomerulonephritis model (SCG/Kj). Apolipoprotein A-2 (ApoA2) has been identified as one of VasSF’s target molecules.

**Methods:**

Here, we tested the potential of anti-apolipoprotein A-2 antibodies (anti-ApoA2) as a new therapeutic drug against KD by examining its effect on a mouse model, in which KD was induced via *Candida albicans* water-soluble fraction (CAWS). CAWS (2 mg/mouse) was injected intraperitoneally into C57BL/6NCrSlc mice for five consecutive days. The incidence and histological severity of vasculitis in CAWS-induced coronary arteritis in mice administered anti-ApoA2 was examined. The following experimental groups were tested: solvent (only PBS (−) injection); anti-ApoA2 antibodies at dosages of 0.05 mg, 0.1 mg, and 0.5 mg/kg/day; human IgG at 0.1 mg/kg/day.

**Results:**

The group treated with anti-ApoA2 0.5 mg/kg/day showed a lower incidence of panvasculitis induced by CAWS, less inflammation of the coronary arteries and aortic roots, and lower levels of serum IL-6, M-CSF, and MIP-1α and 32 cytokines/chemokines compared with those in the solvent group.

**Conclusions:**

The anti-ApoA2 treatment suppressed the development of coronary arteritis in an animal KD model and anti-ApoA2 shows potential as an effective therapeutic candidate for the treatment of KD vasculitis. The use of specific antibodies that display higher vasculitis-suppressing effects, such as anti-ApoA2, may attenuate KD as well as other infectious diseases, with less severe adverse side effects than treatment with IVIg.

## Introduction

Kawasaki disease (KD) is presently garnering worldwide attention as children infected with SARS-CoV-2 exhibit symptoms similar to patients infected with KD [1, 2]. KD is an acute inflammatory illness that affects blood vessels throughout the body during childhood [3]. Coronary arteritis is the most important complication of KD. Murata et al. established a murine systemic vasculitis model using *Candida albicans* (*C. albicans*)-derived substances (CADS) in 1979 [4]. This model demonstrated the role of myeloperoxidase toward coronary artery vasculitis associated with myeloperoxidase-antineutrophil cytoplasmic antibody (MPO-ANCA) production [5]. Later, Nagai-Miura et al. [6] clarified that a *C. albicans* water-soluble fraction (CAWS), eluted from a culture supernatant of *C. albicans* grown in a fully synthetic medium, also induces vasculitis similar to that in Murata’s model. The lesion distribution and histological characteristics of vasculitis in these models was similar to that seen in vasculitis associated with KD. Thus, aortic roots and coronary artery bifurcations in the model are frequently affected by severe inflammatory infiltration, which is caused mainly by neutrophils and macrophages. Although it is believed to be triggered by unidentified infectious pathogens or by uncontrolled immune effects in the case of genetically predisposed children, the causative of KD remains unknown.

High-dose intravenous immunoglobulin (IVIg) treatment remains the first therapeutic choice for KD [7, 8]. Although the presumed mechanism underlying the therapeutic effect of IVIg therapy on KD has been described [9], the exact mechanism remains unknown. Furthermore, 15–20% of patients with KD are IVIg-resistant, and thus require another round of IVIg treatment or adjunctive therapies. IVIg therapy is used not only for KD but also for severe infections and various autoimmune diseases. Although domestic supply of immunoglobulin preparations is secured, the yearly demand for it appears to be increasing.Furthermore, the production of immunoglobulins is expensive. Under such circumstances, preparation of recombinant immunoglobulins is of importance. Based on the fact that previous studies had used human immunoglobulins against CAWS-induced vasculitis to demonstrate the efficacy of IVIg treatment, we hypothesized that the CAWS-induced KD model would be suitable for testing the therapeutic effects of recombinant immunoglobulins [10].

We have been developing recombinant immunoglobulins to circumvent the risks associated with human-derived immunoglobulin production. We produced recombinant immunoglobulins, which contained a polyclonal mixed batch of recombinant single chain fragments of variable IgG regions (hScFv) with VH-CH1 hinge composition [11]. Subsequently, hScFv was injected into a mouse model of spontaneous crescentic glomerulonephritis and anti-neutrophilic cytoplasmic antibody (ANCA)-related vasculitis, SCG/Kj. Injection of hScFv exerted a suppressive effect on the development and production of MPO-ANCA and inflammatory cytokines in the SCG/Kj mice. The hScFv protein mixture showed therapeutic effects at IVIg dosage between 1/10–1/40 in the SCG/Kj mice [11]. Another study also demonstrated that hScFv slightly suppressed vasculitis development in a murine KD model [10]. Moreover, treatment with a single clone of hScFv, named as VasSF, also exerted a suppressive effect on the development of vasculitis in SCG/Kj mice [12]. Molecular MS/MS analysis enabled us to identify VasSF bound apolipoprotein A-2 (ApoA2). SCG/Kj mice which were administered anti-apolipoprotein A-2 antibodies (anti-ApoA2) recovered from vasculitis related renal disorders and showed a decrease in the levels of MPO-ANCA and inflammatory cytokines. As a result, we confirmed that apolipoprotein A-2 was VasSF’s therapeutic target in vasculitis [12].

In the present study, we examined the suppressive effect of anti-ApoA2 on the development of vasculitis with coronary arteritis in a CAWS-induced murine KD model via histological evaluation and the detection of inflammatory cytokines.

## Methods

### Animals

Four-week-old male C57BL/6NCrSlc mice were purchased from Sankyo Labo Service Corporation (Tokyo, Japan). These mice were housed under approved specific pathogen-free conditions in a temperature-controlled environment under a 12-hour light/dark cycle. All animal experiments were performed in accordance with institutional regulations, and in compliance with the Act on Welfare and Management of Animals and the related guidelines in Japan. All the experiments were approved by the Animal Experimentation Ethics and the Genetically Modified Organism Experimental Safety Committees of Teikyo University (permit No. 16–008, 2016).

### Induction of vasculitis

CAWS were utilized to induce vasculitis according to a previously described method [13]. One mg of CAWS suspended in 0.2 mL of Dulbecco’s phosphate-buffered saline (PBS) was injected intraperitoneally into each mouse once a day for 5 consecutive days. The mice were sacrificed via isoflurane inhalation 28 days after the continuous inoculation of CAWS was started.

### Administering of anti-ApoA2 and hIgG to the mice

Starting from the third day after CAWS inoculation, purified Anti-ApoA2 rabbit antibodies (Sigma-Aldrich Inc., St. Louis, MO, USA) and human native immunoglobulin G (hIgG, Nihon Pharmaceutical Co., Ltd., Tokyo Japan) were intraperitoneally administered in the mice for 5 consecutive days,. The dosage of anti-ApoA2 was 0.05, 0.1, and 0.5 mg/kg body weight (kg bw)/day. As a positive control, hIgG was intraperitoneally administered at 0.1 mg/kg bw/day for the 5 consecutive days.

### Experimental groups

Five groups were set up as follows: (i) solvent (only PBS) (*n* = 7); (ii) anti-ApoA2, 0.05 mg/kg/day (*n* = 8); (iii) anti-ApoA2 0.1 mg/kg/day (*n* = 8); (iv) anti-ApoA2 0.5 mg/kg/day (*n* = 8); (v) hIgG, 0.1 mg/kg/day (*n* = 7). C57BL/6NCrSlc mice (*n* = 7) were used as healthy controls.

### Histological evaluation of vasculitis

Histological assessments were conducted in accordance with previously described methods [13, 14]. After the mice were sacrificed, serial sections of the coronary arteries and the aortic roots were stained using the hematoxylin and eosin (HE) staining method. Stained specimens were carefully examined for inflammatory lesions on the vessel wall under a light microscope. Each site was anatomically divided into 5 segments as follows: left coronary artery; right coronary artery; left coronary sinus; right coronary sinus; non-coronary sinus. The degree of inflammation in each segment was assessed using four scores: 0 = no inflammation; 1 = inflammation in the intima (i.e., endoarteritis); 2 = inflammation in the intima and adventitia; 3 = inflammation in all the layers of the vascular wall (i.e., panvasculitis). The total number of segments with a score of 1 or more was defined as the extent of the lesion, while the total score for all the 5 segments was defined as the degree of inflammation in each mouse. Panvasculitis was defined as testing positive for vasculitis. A comparative investigation of the incidence of vasculitis at the coronary artery and aortic root was performed. In order to evaluate the histological severity in the coronary arteries and aortic roots, the extent of the lesions and degree of inflammation were compared among each group.

### Measurement of serum cytokines and chemokines with bio-Plex

Serum cytokine and chemokine levels were quantified using Bio-Plex 200 (Bio-Rad Laboratories, CA, USA), a multiplex cytokine array system, according to the manufacturer’s instructions. The Bio-Plex mouse Cytokine 23-Plex Panel included interleukin (IL)-1α, IL-1β, IL-2, IL-3, IL-4, IL-5, IL-6, IL-9, IL-10, IL-12p40, IL-12p70, IL-13, IL-17, eotaxin, granulocyte colony-stimulating factor (G-CSF), granulocyte macrophage colony-stimulating factor (GM-CSF), IFN-γ, KC/CXCL1, monocyte chemotactic protein-1 (MCP-1), macrophage inflammatory protein-1α (MIP-1α), MIP-1β, regulated on activation normal T cell expressed and secreted (RANTES), and TNF-α. The Bio-Plex mouse Group II panel included IL-15, IL-18, basic fibroblast growth factor (FGF-basic), leukemia inhibitory factor (LIF), M-CSF, monokine induced by gamma interferon (MIG), MIP-2, platelet-derived growth factor-BB (PDGF-BB), and vascular endothelial growth factor (VEGF). Data acquisition and analysis were performed using Bio-Plex Manager software version 5.0.

### Statistics

The incidence of panvasculitis in each group was examined using the chi-square test. Histological severities and cytokine/chemokine levels were analyzed via the Kruskal-Wallis test. If this test found a significant intergroup difference, the Steel-Dwass tests were used for multiple comparisons among experimental groups. Statistical significance was set at *P* < 0.05. All calculations were performed using the statistical software package IBM SPSS v 24.0.

## Results

### Histological observations of vascular lesions in groups treated with anti-ApoA2

**Fig. 1:**
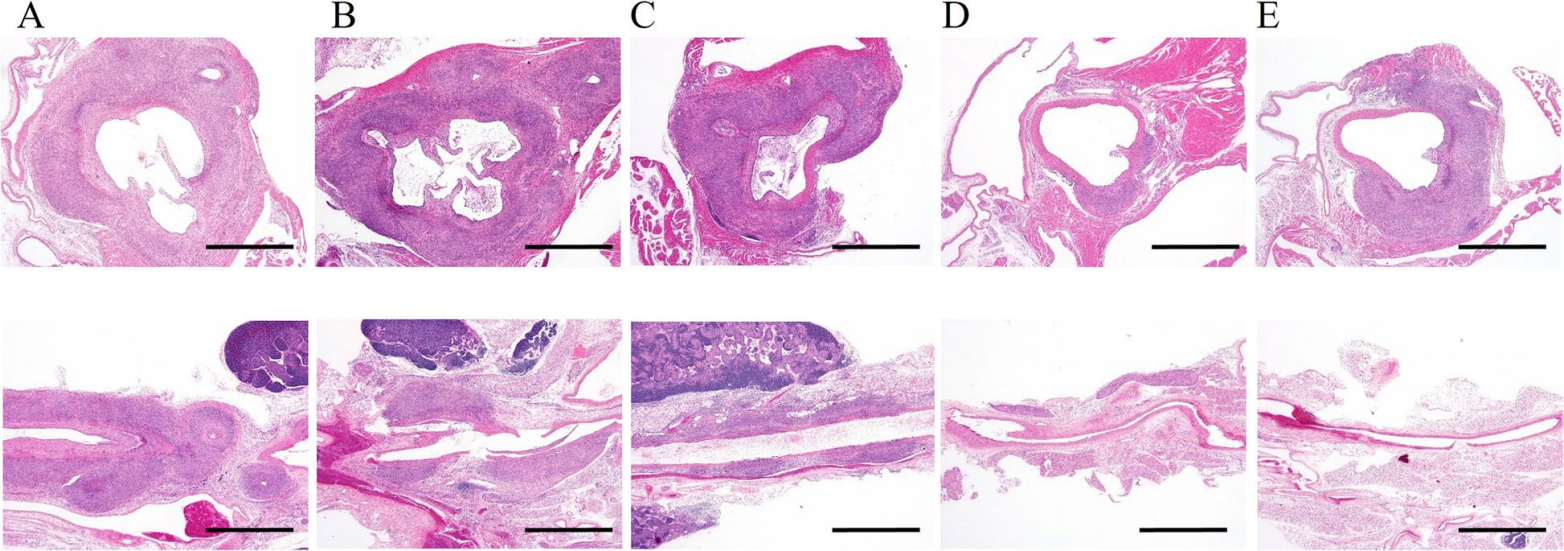
Histology of the vascular lesions. Histological images of coronary arteries, aortic roots (upper row), and abdominal aorta (lower row). H & E staining. Scale bar = 1 mm. **A** Solvent (PBS); **B** anti-ApoA2 0.05 mg/kg/day; **C** anti-ApoA2 0.1 mg/kg/day; **D** anti-ApoA2 0.5 mg/kg/day; **E** hIgG 0.5 mg/kg/day

The histology of arteritis in each experimental group is shown (Fig. 1). In the groups that were administered with the solvent, anti-ApoA2 0.05 mg/kg/day, or anti-ApoA2 0.1 mg/kg/day, severe panvasculitis encompassing the aortic roots, including coronary bifurcations, and abdominal and thoracic aortas was observed (Fig. 1A-C). Inflammatory cells, mainly consisting of neutrophils and histiocytes, accompanied by proliferation of fibroblasts and capillaries were observed in all the layers of the vessel walls. On the other hand, panvasculitis in the aortic roots could not be completely suppressed even in the group administered with anti-ApoA2 0.1 mg/kg/day and hIgG, but the extent of vasculitis was smaller than that in the solvent group. However, many segments without even a small degree of inflammation or with just mild inflammation, such as in endoarteritis, were also observed.

Although panvasculitis was observed in the abdominal aortas of the hIgG-administered group, this was not the case in the abdominal and thoracic aortas of the anti-ApoA2 0.1 mg/kg/day group (Fig. 1D-E). However, when panvasculitis developed in any of the treatment groups, the types and numbers of inflammatory cells infiltrating the lesion were similar to those of the solvent group.

### Decreased incidence of panvasculitis after treatment with anti-ApoA2

**Fig. 2:**
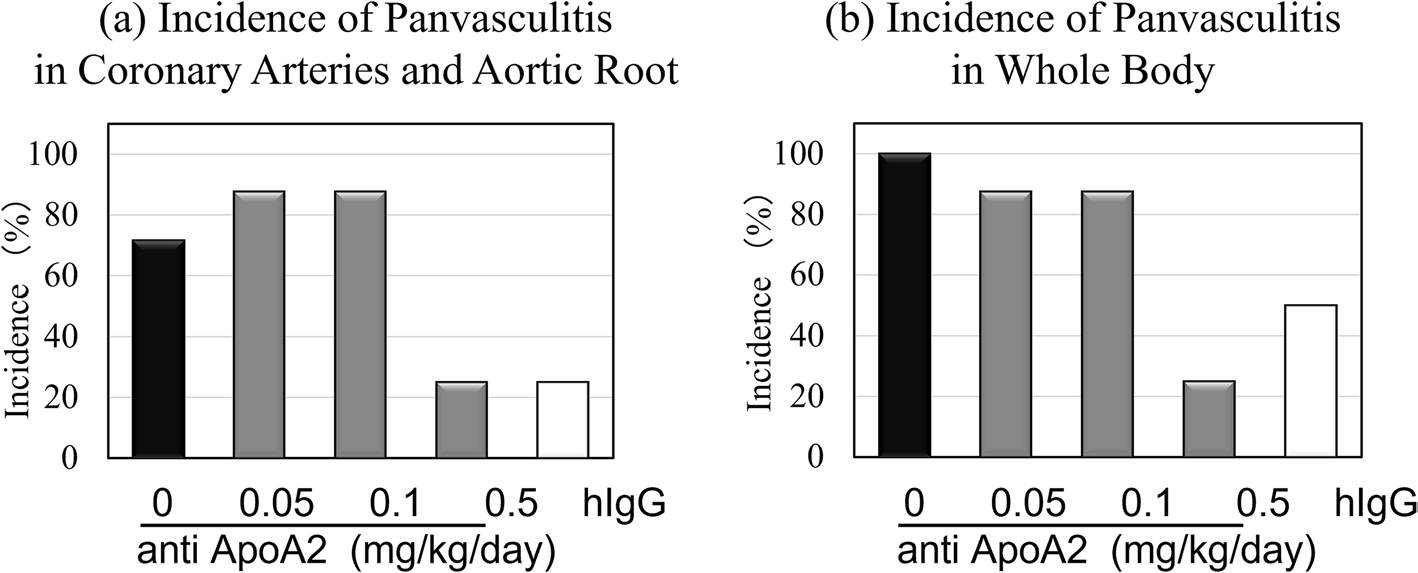
Effects of anti-ApoA and hIgG on incidence of CAWS-induced panvasculitis. **a** Incidence of Panvasculitis in Coronary Arteries and Aortic Root. **b** Incidence of Panvasculitis in Whole Body. Bar graph showing the incidence of panvasculitis. Black bar: solvent; dark gray bars: treatment with anti-ApoA2; white bar: treatment with hIgG 0.1 mg/kg/day

We determined the incidence of panvasculitis in the coronary artery and aortic roots in each group via histological observation. Panvasculitis incidence in the different groups subjected to different treatments was 71.4% (5/7 mice) for solvent, 87.5% (7/8 mice) for anti-ApoA2 0.05 mg/kg/day, 75% (6/8 mice) for anti-ApoA2 0.1 mg/kg/day, 25% (2/8 mice) for anti-ApoA2 0.05 mg/kg/day, and 28.6% (2/7 mice) for hIgG 0.1 mg/kg/day (Fig. 2a). Groups administered with anti-ApoA2 0.5 mg/kg/day and hIgG 0.1 mg/kg/day showed lower incidence than that in the solvent group. Moreover, the incidence of whole body panvasculitis, based on the histological observations of the lung, spleen, kidney, heart and skin for each group was as follows: 100% (7/7 mice) for solvent; 87.5% (7/8 mice) for anti-ApoA2 0.05 mg/kg/day; 87.5% (7/8 mice) for anti-ApoA2 0.1 mg/kg/day: 25.0% (2/8 mice) for anti-ApoA2 0.5 mg/kg/day; 57.1% (4/7 mice) for hIgG 0.1 mg/kg/day (Fig. 2b). Panvasculitis occurred in all the groups, but its incidence in the group treated with anti-ApoA2 0.5 mg/kg/day tended to be particularly lower than that in the solvent group. We specifically observed panvasculitis in some parts of the abdominal and thoracic aortas.

### Decreased inflammation in the 0.5 mg/kg/day anti-ApoA2 treatment group

**Fig. 3:**
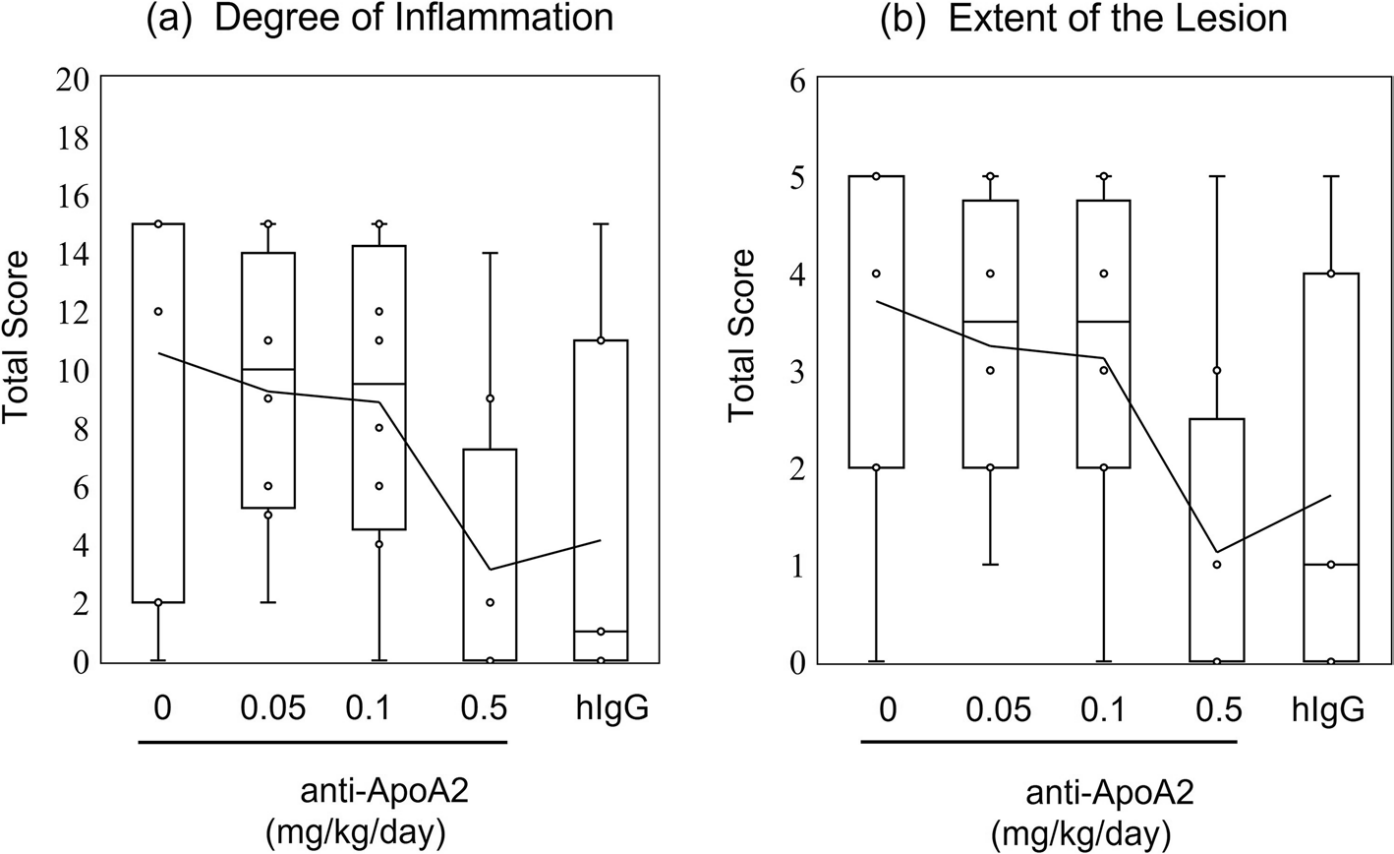
Histological severity of vasculitis. **a** Degree of Inflammation. **b** Extent of the Lesion. The number of segments involved indicates: **a** the size of the lesion and inflammation score; **b** the degree of inflammatory cell infiltration

The degree of inflammation in each group, as indicated by the total score for all the 5 segments in each mouse, was as follows: 10.57 ± 2.52 for solvent; 9.25 ± 1.66 for anti-ApoA2 0.05 mg/kg/day; 8.88 ± 1.89 for anti-ApoA2 0.01 mg/kg/day; 3.13 ± 1.90 for anti-ApoA2 0.5 mg/kg/day; 4.14 ± 2.33 for hIgG 0.1 mg/kg/day (Fig. 3a). The degree of inflammation in groups treated with anti-ApoA2 0.5 mg/kg/day was significantly lower than that in the solvent group (*P* < 0.05), while that in all the treatment groups tended to be lower than that in the solvent group.

### Effect of anti-ApoA2 treatment on the extent of the lesion

The extent of the lesion, as indicated by the total number of lesion segments per 5 segments in each mouse, in each group were as follows: 3.71 ± 0.75 for solvent: 3.25 ± 0.53 for anti-ApoA2 0.05 mg/kg/day; 3.13 ± 0.61 for anti-ApoA2 0.01 mg/kg/day; 1.13 ± 0.67 for anti-ApoA2 0.5 mg/kg/day; 1.71 ± 0.75 for hIgG 0.1 mg/kg/day (Fig. 3b). The extent of the lesion in the treatment groups tended to be smaller than that of the solvent group.

### Cytokines/chemokines in serum

**Fig. 4:**
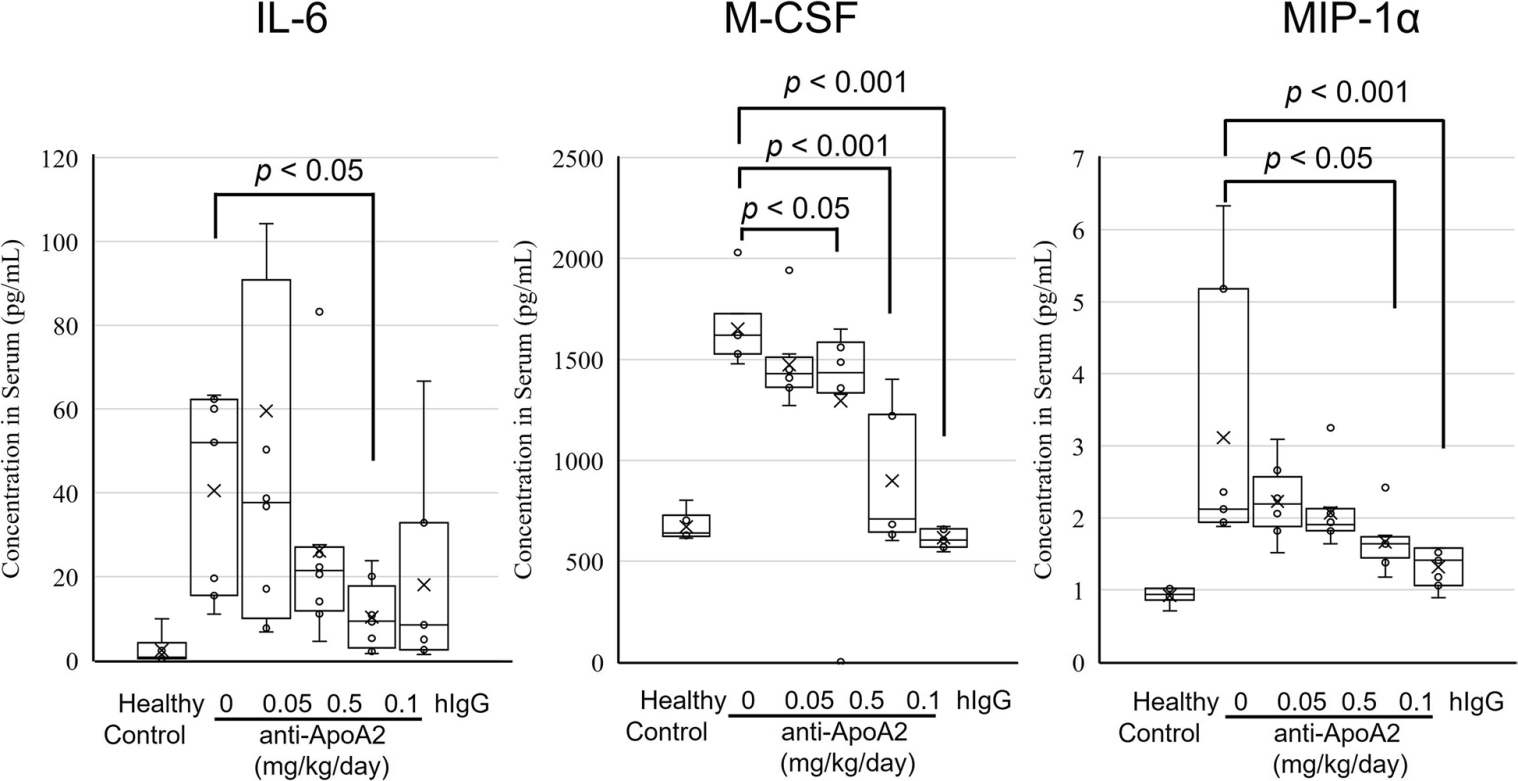
Reduction of cytokines by treatment with anti-ApoA2. Cytokines were measured in serum from healthy control and therapeutic mice

We made the comparison between the cytokine/chemokine profiles of healthy controls and KD models. The levels of the 24 cytokines and chemokines, IL-1β, IL-2, IL-3, IL-4, IL-5, IL-6, IL-9, IL-12p40, IL-13, GM-CSF, IFN-γ, KC, MCP-1, MIP-1α, MIP-1β, TNF-α, IL-15, IL-18, FGFbasic, LIF, M-CSF, MIG, MIP-2, and VEGF in the plasma of the KD mouse model induced by CAWS were significantly higher than those in the plasma of the healthy control. We focused on these 24 cytokine/chemokines in the treatment groups. The levels of inflammatory cytokines and chemokines, IL-1β, IL-2, IL-4, IL-5, IL-6, IL-12p40, IL-13, MCP-1, MIP-1α, TNF-α, LIF, M-CSF, MIG, MIP-2, and VEGF, in the anti-ApoA2 treatment groups tended to be lesser than those of the solvent group. However, the levels of the 24 cytokines/chemokines in the anti-ApoA2 0.05 and 0.1 mg/kg/day treatment groups failed to show statistically significant suppression. Nevertheless, the levels of inflammatory cytokines, such as IL-6 (*P* < 0.05), M-CSF (*P* < 0.001), and MIP-1α (*P* < 0.05) in the anti-ApoA2 0.5 mg/kg/day treatment group were significantly suppressed (Fig. 4). Furthermore, hIgG treatment group, used as the positive control, also significantly suppressed the cytokine levels of M-CSF (*P* < 0.001) and MIP-1α (*P* < 0.001).

## Discussion

Our histological evaluation of vascular lesions and the measurement of serum cytokine/chemokine levels in the mouse KD model revealed that anti-ApoA2 treatment suppressed the development of coronary arteritis in a mouse model of KD vasculitis in a dose-dependent manner. Thus, anti-apoA2 treatment showed potential as an effective therapeutic candidate for KD. Histological observations indicated that anti-ApoA2 induced histological changes in CAWS-induced vasculitis in a dose-dependent manner. Under these conditions, lesion distribution as well as the histological aspects of vasculitis in the CAWS-induced mouse models were similar to those of vasculitis in patients with KD [14]. In addition, the aortic roots and bifurcations of coronary arteries were affected by severe infiltration of inflammatory cells, mainly neutrophils and macrophages. Although panvasculitis was observed in all the groups in the present study, treatment with anti-ApoA2 reduced its incidence, leaving only partial inflammation in the aortic roots and abdominal aortas. Administering high doses of IgG adversely affects renal function to some degree [15]. Therefore, serious adverse events associated with the use of IVIg may be avoided by using specific therapeutic drugs with a higher vasculitis-suppressing effect instead. However, many mice in the anti-ApoA2 treatment group showed ventricular hypertrophy on both the sides than those in the hIgG treatment group, indicating that this may be one of the side effects of anti-ApoA2 treatment.

In the present study, the quantitative evaluation of vascular inflammation indicated that administering anti-ApoA2 suppressed the incidence, scope, and degree of inflammation in the coronary arteries and aortic roots. This was in line with previously reported beneficial effects of anti-ApoA2, such as effective recovery from renal disorders and decreased levels of MPO-ANCA and inflammatory cytokines in SCG/Kj mice models of MPO-ANCA-associated vasculitis (MAAV) [12]. ApoA2 may be associated with a similar risk in mouse vasculitis models because it is one of the target antigens of VasSF, which has been developed as a novel antibody drug, derived from IgG, for vasculitis in SCG/Kj mouse [12].

Furthermore, we observed that the serum levels of the cytokines/chemokines IL-6, M-CSF, and MIP-1α were drastically decreased by treatment with anti-ApoA2 in mice. These results were similar to those in studies on humans, where the levels of IL-6 and M-CSF were significantly decreased, whereas those of MIP-1α were also decreased, but not significantly. By contrast, the levels of IL-6, M-CSF, and MIP-1α were all significantly decreased after treatment with IVIg. Suppression of M-CSF decreases obstacles because M-CSF produced in endothelial cells induces the recruitment of macrophages to enhance inflammation [16, 17]. In addition, IL-6 plays a key role in restraining inflammatory disease [18]. Therefore, a decrease in the levels of both these cytokines suppresses the inflammation in blood vessels. A similar reduction in serum IL-6 levels has been reported in patients with KD who respond to treatment with IVIg [19]. M-CSF inhibits the migration of monocytes and macrophages into the vascular cavity. Such inhibition promotes the formation of early lesions of arteriosclerosis indicating that M-CSF may induce lesion formation. The results of the present study suggest that administering anti-ApoA2 suppressed lesion formation during the early stages. MIP-1α-induced inflammatory responses are similarly decreased by the immune modulating drug, mizoribine, which is administered to patients with KD who do not respond to IVIg treatment [20]. These findings indicated that anti-ApoA2 treatment suppresses cytokine/chemokine response associated with vasculitis. Although most patients with KD subjected to standard care involving high-dose IVIg have shown excellent response [21, 22], 15–20% of these patients do not respond to the first IVIg treatment [22]. In addition, the use of IVIg for the purpose of treating serious infectious and autoimmune diseases is increasing year by year. Thus, the need to develop new therapeutic agents is urgently felt. The current study showed that anti-ApoA2 may act as an effective therapeutic agent for treating patients with KD. Antibody based drugs, such as infliximab (an anti-human tumor necrosis factor (TNF)-α monoclonal antibody [23, 24]), cytokine antagonists (anakinra, interleukin 1 receptor antagonist, IL-1RA [25]), and immunosuppressants (e.g., mizoribin [20] and sivelestat [26]) have recently been suggested as therapeutic agents for patients with IVIg-resistant KD. As the suppression of TNF-α and IL-1RA diminishes the host response to infections, concerns have been raised regarding the safety of administering infliximab to patients receiving live viral vaccines. However, it remains the drug of choice for patients with IVIg-resistant KD [27]. Although anti-cytokine therapy effectively suppresses inflammation, it does not treat the root cause of the symptoms. Therefore, even if clinical remission is induced by anti-cytokine therapy, the risk for relapse remains.

Our results suggest that ApoA2, which is involved in vasculitis and is the second most abundant protein in high-density lipoprotein (HDL), stabilizes the structure of HDL via its association with lipids. Lipid profiles of patients with KD exhibit significant abnormalities [28]. In these patients, mean plasma concentrations of total and HDL cholesterol are profoundly reduced during the earliest days of illness [29, 30]. However, total cholesterol levels rapidly return to normal, whereas that of HDL cholesterol recover more slowly following the onset of KD. Lipid findings pertaining to the early clinical course of KD are consistent with those delineated in various acute infections [31, 32]. Furthermore, a high concentration of blood MPO, produced by the MPO-HOCL system, facilitates the formation of the ApoA1-ApoA2 heterodimer in HDL [33]. In addition, clinical studies strongly indicate that the plasma concentrations of MPO are associated with the occurrence and severity of coronary artery diseases [5, 34]; patients with coronary artery diseases exhibit significantly high MPO concentrations. High levels of blood MPO and inflamed blood vessels, which are observed in patients with KD indicate high oxidative stress [35]. In mice administered with anti-ApoA2, the antibody may interact with the increasing ApoA1-ApoA2 heterodimer. This molecular interaction may suppress the decrease in anti-inflammatory activity, suggesting that anti-ApoA2 may be therapeutically effective not only in vasculitis, but also in various other diseases, such as cardiovascular and infectious diseases. From this study, it is unclear how ApoA2 is involved in the development and pathogenesis of KD. However, it has been reported that oxidized phospholipids and apolipoproteins, which are also associated with arteriosclerosis and coronary arteriosclerosis, are involved in the pathogenesis and progression of coronary artery lesions in KD [36]. ApoA2 may also be one of the factors causing intense inflammation. In near future, we will investigate how ApoA2 is involved in the pathogenesis of KD.

## Conclusions

The present study used a mouse model of CAWS-induced vasculitis to demonstrate that anti-ApoA2 shows potential as an effective therapeutic candidate for the treatment of KD vasculitis. The use of specific antibodies that display higher vasculitis-suppressing effects, such as anti-ApoA2, may attenuate KD as well as other infectious diseases, with less severe adverse side effects than treatment with IVIg. Furthermore, anti-ApoA2 shows potential not only as a therapeutic agent but also as a tool that may be useful for elucidating the etiology and pathophysiology of KD and vasculitis.

## Data Availability

Not applicable.

## Abbreviations

anti-ApoA2: Apolipoprotein A-II antibody
CAWS: *Candida albicans* water-soluble fraction
hIgG: Human native immunoglobulin G
hScFv: Human single chain fragment of variable region,
HE: Hematoxylin and eosin,
IVIg: Intravenous immunoglobulin
KD: Kawasaki disease
